# Preliminary Microbiological Tests of S-Carvone and Geraniol and Selected Derivatives of These Compounds That May Be Formed in the Processes of Isomerization and Oxidation

**DOI:** 10.3390/molecules27207012

**Published:** 2022-10-18

**Authors:** Agnieszka Wróblewska, Anna Fajdek-Bieda, Agata Markowska-Szczupak, Monika Radkowska

**Affiliations:** 1 Department of Catalytic and Sorbent Materials Engineering, Faculty of Chemical Technology and Engineering, West Pomeranian University of Technology in Szczecin, Piastów Ave. 42, 71-065 Szczecin, Poland; 2 Jakub’s from Paradyż Academy in Gorzów Wielkopolski, Teatralna 25, 66-400 Gorzów Wielkopolski, Poland; 3 Department of Chemical and Process Engineering, Faculty of Chemical Technology and Engineering, West Pomeranian University of Technology in Szczecin, Piastów Ave. 42, 71-065 Szczecin, Poland

**Keywords:** natural preservatives, S-carvone, carvacrol, geraniol, nerol, linalool, citral

## Abstract

This work presents a literature review on the biological activity of S-carvone, geraniol and derivatives of these compounds, which are formed in the process of isomerization (during the process of geraniol isomerization, oxidation products of this compound are also obtained). Moreover, this work presents preliminary microbiological tests of creams with the addition of these biologically active compounds: S-carvone, geraniol, carvacrol (an S-carvone isomerization product), nerol (a geraniol isomerization product), linalool (a geraniol isomerization product) and citral (a geraniol oxidation product). Because the post-reaction mixture obtained after the S-carvone isomerization has a relatively simple composition, it was also added to creams and tested without isolating pure compounds. This may be a cheaper alternative to creams prepared with the addition of pure compounds. The mixture obtained after the geraniol isomerization process has a very complex composition; therefore, only compounds with the lowest molecular weight and are easily commercially available were selected for studies. The content of the tested compounds in the creams ranged from 0.5 to 3 wet%. The following microorganisms were selected for microbiological tests: the Gram-negative bacterium *Escherichia coli* K12, the Gram-positive bacterium *Staphylococcus epidermidis*, and the fungi *Candida albicans, Trichophyton rubrum, Aspergillus niger*, and *Penicillium chrysogenum.* A content of 3% carvacrol, nerol, geraniol and citral inhibited the growth of *E. coli*, and attenuated the growth of *C. albicans and T. rubrum*. On the other hand, 3% carvacrol and citral only poorly attenuated the growth of the mould fungi *P. chrysogenum* and *A. niger.*

## 1. Introduction

Despite the significant progress in medicine and pharmacy in recent decades, traditional therapeutic strategies, which are used to treat virial, bacterial or fungal diseases, are often unsatisfactory and cause many side effects (e.g., change in the microbiome) [1]. Another major problem is the emergence of resistance of bacteria to many of the antibiotics that have been used so far [2]. Therefore, the interest in compounds of plant origin as active compounds that can be used in the treatment of many diseases, including infectious diseases, is growing. An example of such compounds are those belonging to the terpene group. Terpenes and their derivatives are a huge group of natural organic compounds that are part of many essential oils. Numerous studies have confirmed that essential oils and their terpene components have a wide range of biological activity in vitro. It has been proven that they have antibacterial, antiviral, antifungal and antiparasitic properties. In addition, they exhibit anti-inflammatory activity and stimulate the immune system [3]. In this work, particular attention was paid to the biological activity of S-carvone, geraniol and derivatives of these compounds which are formed in the process of isomerization (during the process of geraniol isomerization, oxidation products of this compound are also formed), such as carvacrol the (main product of S-carvone isomerization), nerol (a geraniol isomerization product), linalool (a geraniol isomerization product) and citral (a geraniol oxidation product). These compounds were selected for microbiological tests in the experimental part of this study and were added to the creams described in this article. We also used, as additives to the creams, the post-reaction mixtures obtained after the isomerization of S-carvone and geraniol, from which the catalyst was previously separated. These mixtures were solvent-free and could be a cheaper alternative to pure compounds added to creams. Moreover, the addition of post-reaction mixtures may be associated with the occurrence of a synergistic effect of their components. The mixture obtained after isomerization of S-carvone only contained unreacted S-carvone, carvacrol and very small amounts of other products and, therefore, it was used for preparation of the creams. As the post-reaction mixture obtained after the isomerization of geraniol had too complicated a composition, its addition to creams was eventually abandoned.

A valuable source of S-carvone is cumin. Cumin (*Carum carvi* L.) is a biennial plant belonging to the celery family. Cumin seeds contain 2–8% of essential oils, which contain two main compounds: S-carvone (50–70%) and R-limonene (25–30%) [4,5]. Ingredients contained in cumin cause the diastolic effect of smooth muscles of the gastrointestinal tract, which prevents colic. In addition, consumption of cumin stimulates the secretion of digestive juices. Moreover, cumin has anti-inflammatory properties and prevents flatulence [6]. The properties of cumin result in its use in the diet of humans and farm animals. In the case of food products, cumin seeds are used as additives to bread, cheese, sauerkraut, candies, meat products, sauces and liqueurs. Cumin is also a valuable raw material for medicine. Studies performed in rats showed that cumin administered orally inhibits the development of colorectal cancer [7,8].

S-carvone is the main component of cumin oil. This compound is characterized by high biological activity. According Hartmans et al., S-carvone can be used as a natural inhibitor of germination. It exhibits the same, or even better properties, than preparations commonly used in potato storage [9]. The antibacterial properties of carvone have been investigated against *Escherichia coli* and *Staphylococcus aureus*. In these studies, the carrier for this compound were nanoparticles from poly-lactic glycolic acid (PLGA). The minimum inhibitory concentration (MIC) of nanoparticles filled with carvone was much lower in the case of *S. aureus* (182 mg/mL), belonging to Gram-positive bacteria, than in the case of *E. coli* (374 mg/mL), which are Gram-negative bacteria [10]. In other studies, carvone has been used as an antibacterial coating in medical devices. These studies showed that carvone formed a hydrophobic bacterial coating that, even after 10 days of aging in the air, maintained a static contact angle with water amounting 78°, and stability for 24 h during culture in Luria-Bertani (LB) liquid media broth. An effective reduction in the number of *E. coli* and *S. aureus* (by 86% and 84%, respectively) was observed during these studies [11]. *Candida albicans* is an opportunistic pathogenic yeast that occurs naturally in the human gut. This species is the most important pathogen causing human candidiasis, which is especially dangerous for people with a weakened or compromised immune system. S-carvone has proven to be a potential therapeutic agent in the treatment of yeast infections caused by *C. albicans*. It was observed that this monoterpene inhibits the transformation of *C. albicans* into the filamentous form that is associated with the pathogenicity of this fungus [12]. Carvone has also been tested for immunomodulatory effects, and it was shown that it stimulates the immune system to regulate the body’s immune response. Immunosuppression is one of the huge obstacles faced by cancer patients during chemotherapy and radiation therapy. Human immunity depends, among others, on the amount of white blood cells. The use of monoterpenes in these studies increased the number of white blood cells in mice. In addition, it was shown that feeding animals with carvone caused an increase in the number of bone marrow cells [13].

As mentioned above, the main product of S-carvone isomerization is carvacrol [14]. Studies on carvacrol have shown its anticancer properties. Carvacrol was effective in reducing the proliferation and apoptosis of colon cancer cell lines (e.g., HCT116) [15]. This compound also showed a positive effect on liver regeneration [16,17]. It was found that carvacrol has antimicrobial activity against two *Vibro cholerae* strains inoculated in carrot juice. In addition, cymene, which did not show antimicrobial activity against these bacteria, supported the carvacrol inhibitory effect [18]. *Staphylococci* are other pathogenic bacteria not resistant to carvacrol. It was observed that after 24 h and without the addition of carvacrol, an *S. aureus* 815 biofilm matrix was formed, and carvacrol at a minimal inhibitory concentration (0.25 MIC) caused destruction of this bacterial biofilm (only single cells were observed) [19]. Currently, medical treatments are increasingly dealing with antibiotic-resistant bacteria. Such bacteria are strains capable of producing β-lactamase, which is responsible for the resistance of these microorganisms to antibiotics. The effects of carvacrol on beta-lactamase *Escherichia coli* (EBLS) isolated from the peritoneal fluid of patients with urinary tract infections have been investigated. These studies showed that carvacrol is effective against EBLS strains, which are responsible for urinary tract infections in women. In addition, this compound affected the motility of these bacteria [20]. There have been numerous studies on the antifungal activity of carvacrol. For example, the antifungal activity of carvacrol against *C. albicans* has been evaluated. After 48 h of incubation with carvacrol, the lowest growth inhibitory concentration and the minimum fungicidal concentration of *C. albicans* were determined as 0.001% (*v*/*v*). Based on these tests, it was concluded that carvacrol has potential use for the local treatment of *C. albicans* infection [21,22,23].

Geraniol is the terpene alcohol that occurs in the essential oils of several aromatic plants. This compound has a characteristic rose-like smell. Geraniol has many applications in the flavor and fragrance industries [24]. It exhibits insecticidal and repellent properties and is used as a natural pest control agent, exhibiting low mammalian toxicity [25,26]. Moreover, geraniol has acaricidal activities against the storage food mite *Tyrophagus putrescentiae*, and a significant anthelminthic activity against the root-knot nematode (*Meloidogyne incognita*) [24]. It also represents a new class of chemoprevention agents for cancer (murine leukemia, hepatoma, melanoma and colon cell) [24,25,26], and has activity against pancreatic and breast cancers [24]. This terpene compound also has antimicrobial, antioxidant, and anti-inflammatory activity [24,25,26]. Geraniol is active against *E. coli* (with the bactericidal activity value of BA50 0.15), against *L. monocytogenes* (with the bactericidal activity value of BA50 0.28) and *S. enterica* (with the bactericidal activity value of BA50 0.15) [24,25]. Moreover, geraniol used in the gaseous state has antibacterial activity against respiratory tract pathogens, including *Haemophilus influenzae*, *Streptococcus pneumoniae*, *S. pyogenes* and *Staphylococcus aureus* [24,25]. Antitubercular activity of geraniol against *Mycobacterium tuberculosis* has been observed [24,25], and other studies have confirmed that the antifungal action of palmarose oil against *S. cerevisiae* is mainly attributable to geraniol, which is a component of this oil [24]. Carvacrol, geraniol and thymol were able to significantly reduce biofilm development of *Candida albicans* strain [24,25], and in other studies, palmarose oil and geraniol were both found to inhibit *Cryptococcus neoformans*, a fungus causing infection during the last stages of AIDS [24,25]. Studies on the antioxidant activity of geraniol with a standard antioxidant (Trolox) showed scavenging activities of this compound against the DPPH radical (87.7%, 235.9 mg of Trolox equiv/mL) [24]. Investigations on the immunosuppressive properties of geraniol, using in vitro lymphocyte proliferation assays and an in vivo rat cardiac allograft transplant model, have also been performed. These results revealed that geraniol can prevent acute allograft rejection [24,25]. Transdermal drug delivery systems represent a novel therapeutic approach to the delivery of pharmaceuticals, and geraniol can be used as a penetration enhancer in transdermal drug delivery systems [24]. Another study revealed that addition of tetrahydrogeraniol in a gel containing 5-fluorouracil markedly enhanced 5-fluorouracil permeability [24].

The process of isomerization of geraniol is a very complicated, as it also involves oxidation, dimerization, fragmentation, dehydration and cyclization [27]. Therefore, for studies involving creams, we decided to use only three derivatives of geraniol: two isomerization products (nerol and linalool) and one oxidation product (citral). Application of the post-reaction mixture after isomerization of geraniol can make the interpretation of results difficult.

Linalool is an unsaturated aliphatic alcohol belonging to the terpene group, and has a lily of the valley smell. It is naturally obtained from essential oils as such as those from coriander or orange. Linalool has many applications in the cosmetics industry as a fragrance and is also used in perfume industry [28,29,30,31,32,33]. Linalool and essential oils rich in this terpene have various biological activities, such as antimicrobial, anti-inflammatory, antitumor and antioxidant activities. Linalool, when used at a concentration of 0.1% (*v*/*v*), exhibits antimicrobial activity against different microorganisms (*Staphylococcus aureus*, *Bacillus subtilis*, *Escherichia coli*, *Pasteurella multocida*), and is more active against Gram-positive bacteria compared to Gram-negative bacteria. This compound also shows remarkable activity against periodontopathic and cariogenic bacteria. Linalool minimum inhibitory concentration and minimum bactericidal concentration values range from 0.1 to 1.6 mg/mL. Moreover, linalool isolated from coriander acts antifungally on oral *Candida* isolates in patients with dental problems, as well as *Candida albicans* and non-albicans Candida (NAC) clinical isolates with differential sensitivities to fluconazole [32,33]. In various experimental models of inflammation, the linalool R-enantiomer and a racemate of linalool acted anti-oedematously limiting the inflammatory response [33]. Linalool has antiproliferative activity against a broad spectrum of carcinoma cells, among others, including multidrug resistant human breast adenocarcinoma cells [32,33]. In hematopoietic malignancies, linalool does not affect the development of normal hematopoietic cells even at a cytotoxic concentration of 130 μM. However, the antitumor potential of linalool is limited because it must be used in high doses during tests performed in vivo [32,33]. Aromatherapy specialists use linalool for treatment of anxiety symptoms, and the anxiolytic effect of linalool has been confirmed by many studies. For example, linalool-exposed mice showed less aggression and less anxiety, allowing for easier social interaction [28,29,30,31,32,33]. Recent studies investigated linalool as a treatment for Alzheimer’s disease in a mouse model. Linalool-treated mice showed increase in cognitive and emotional functions [28,29,30,31]. In agriculture, linalool is used as a fumigant, i.e., as a pest repellent [33].

Another compound obtained in the process of geraniol isomerization is nerol. It has a rose-like aroma, which is more pleasant and delicate than the aroma of geraniol, which is a geometric isomer of nerol. It occurs naturally in bergamot and neroli oils. Nerol is a much more expensive compound than geraniol, which is why it is used in perfumery to prepare more expensive and more luxurious fragrance compositions [34]. Moreover, it is a component in decorative cosmetics, shampoos, toilet soaps, and in non-cosmetic products such as household cleaners and detergents [35]. Neroli oil has valuable healing properties. It has an antiseptic effect and is used in compresses to relieve the symptoms associated with ringworm and inflammations of the skin. Neroli oil, due to its antidepressant properties, is used in aromatherapy to improve mental health, soothe nerves, as a sedative, to alleviate anxiety and to strengthen the mind. Neroli oil is used for massage, contains vitamin P, seals capillary walls and supports the action of vitamin C. In addition, it improves blood circulation and relaxes muscles. Neroli oil helps to deal with menopausal and premenstrual syndrome [35]. The neuropharmacological properties of nerol in mice have been studied [36]. Nerol showed antimicrobial activity against six species of bacteria (both Gram-positive and Gram-negative), two species of yeasts and three species of mould fungi. It has pronounced antibacterial activity, especially against Gram-negative *Pseudomonas aeruginosa*. Moreover, nerol showed very strong antifungal activity in an agar-well-diffusion test compared to a standard antibiotic (nystatin) [37,38].

Citral also belongs to the terpene group. It has an intense lemon scent. This compound is the component of many essential oils and, among others, is found in lemon oil, lemongrass oil (from lemongrass) (80% citral), and in oils obtained from the tropical plant *Backhousia citriodora* (lemon myrtle; 90% citral), lemon verbena (30–35% citrale), lemon balm and orange. Citral is used in perfume and in the food industry as a fragrance. It is also used as a flavor enhancer. Citral has antifungal, antibacterial, antioxidant and ant-inflammatory properties. Studies performed in vitro showed its anticancer activity. Citral inhibited growth of the human breast cancer cell line MCF-7, and also showed a beneficial effect in patients with B cell lymphoma treated with chemotherapy [39,40]. Moreover, it inhibited cell viability, proliferation, and the clonogenic potential of prostate cancer cells [41]. Citral can be also used as an efficient painkiller [40]. During oral administration, citral exhibited a significant gastro-protective action against ulcers induced by indomethacin as anti-inflammatory drug [40]. Studies have shown that citral can protect IEC-6 cells against aspirin-induced oxidative stress [40]. Espina et al. [41] investigated the effect of carvacrol and citral on *L. monocytogenes* (EGD-e), *S. aureus* (SC-01) and *E. coli* (MG1655) bacteria. It was found that both citral and carvacrol caused a decline in the number of bacterial cells, and reduced the biofilms formation by all three species. α- and β-citral showed antibacterial activity against both Gram-positive and Gram-negative bacteria [42]. Experiments to investigate the antimicrobial effect of citral on *Y. enterocolitica* have also been performed. The minimum inhibitory concentration and minimum bactericidal concentration of citral against *Y. enterocolitica* ATCC 23715 were 0.2 and 0.4 mg/mL, respectively [43]. Citral also inhibited the growth of *C. albicans* [44]. Cai et al. [45] conducted studies on the antifungal activity of citral, limonene and eugenol against *Zygosaccharomyces rouxii*. All three compounds showed strong antifungal activity against *Z. rouxii*. The minimum inhibitory concentrations of citral, limonene and eugenol were 0.188, 0.75 and 0.4 μL/mL, respectively, whereas the minimum fungicidal concentrations of these compounds were 0.375, 3 and 0.8 μL/mL, respectively.

The main purpose of our work was to present a general overview of literature reports on the biological activity of S-carvone, geraniol and derivatives of these two compounds obtained during isomerization processes (during the process of geraniol isomerization, oxidation products of this compound are also obtained), as well as to perform preliminary microbiological tests of creams containing these compounds. The tests were intended to determine whether such creams could be used to treat various skin diseases. The studied derivatives of S-carvone and geraniol were carvacrol (an S-carvone isomerization product) and nerol (a geraniol isomerization product), linalool (a geraniol isomerization product) and citral (a geraniol oxidation product). Another important goal of our work was to check whether the post-reaction mixtures obtained after S-carvone and geraniol isomerization processes could be used as additive to creams (after separating the catalyst first) without separating them into individual components. The use of post-reaction mixtures instead of pure products would significantly reduce the cost of cream preparation. It was found that the post-reaction mixture obtained after S-carvone isomerization process is not a complicated mixture in terms of composition, and can be added to creams and subjected to microbiological tests. However, the post-reaction mixture obtained after the geraniol isomerization process was a very complex mixture in terms of composition and, in this case, only compounds with the lowest molecular weight and which are easily commercially available were selected for the studies (the complicated composition could affect results and make their interpretation difficult). The content of the tested compounds ranged from 0.5 to 3 wt%. We investigated whether these creams, in addition to being cosmetic, can also have a healing effect on the skin and be used to treat cuts, allergies, eczema or atopic dermatitis. Moreover, the main objective was to assess whether S-carvone and geraniol, and selected derivatives of these compounds, can be used as ingredients of ointments with antimicrobial activities to treat skin diseases or to reduce the risk of microbial growth leading to product spoilage and the loss of product performance.

## 2. Materials and Methods

### 2.1. Preparation of Therapeutic Creams

In the preparation of creams the following raw materials were used: commercially available food grade sunflower oil, white bee wax (EcoFlores company, Nowy Targ, Poland), urea (Zrób Sobie Krem Kosmetyki Naturalne Katarzyna Damętka company, Prochowice, Poland), allantoin (Zrób Sobie Krem Kosmetyki Naturalne Katarzyna Damętka company, Prochowice, Poland), S-carvone (96%, Sigma, Poznań, Poland), carvacrol (98%, Sigma, Poznań, Poland), geraniol (98%, Sigma, Poznań, Poland), nerol (97%, Sigma, Poznań, Poland), linalool (97%, Sigma, Poznań, Poland), and citral (95%, Sigma, Poznań, Poland).

**Figure 1:**
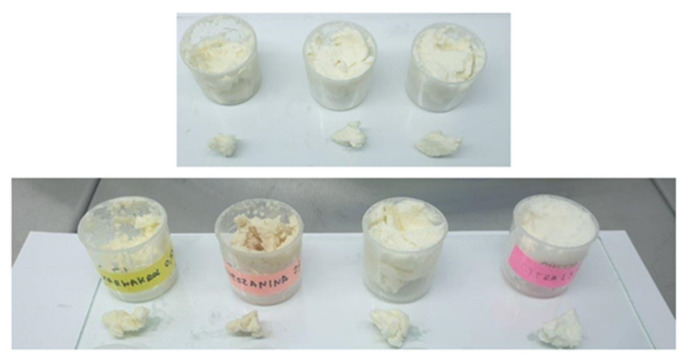
Pictures of prepared creams with the addition of (from the left): carvacrol, the mixture after isomerization of S-carvone, geraniol and citral.

Creams were prepared in the laboratory of Department of Catalytic and Sorbent Materials Engineering West Pomeranian University of Technology in Szczecin. To prepare the creams, an oil phase consisting of 12.5 g safflower oil and 3 g of beeswax was weighed. Next, the water phase with the following composition was weighed: 3.5 g of urea, 0.5 g of allantoin and 21.25 g of water. Active compounds (S-carvone, carvacrol, geraniol, nerol, linalool and citral), or a mixture after the isomerization of S-carvone, were added to the oil phase at concentrations of 0.5, 1.0, 2.0, and 3.0 wt%. Beakers with the water phase and the oil phase were placed in a water bath at a temperature of 80 °C. After dissolving the oil phase ingredients, the beakers were taken out of the bath and the oil phase was added to the water with intense mixing. The mixture was stirred until it reached a homogeneous creamy consistency. Figure 1 presents examples of the prepared creams.

### 2.2. Preparation of the Post-Reaction Mixture after Isomerization of S-Carvone for Application in Creams

Studies on isomerization were performed in the laboratory of Department of Catalytic and Sorbent Materials Engineering West Pomeranian University of Technology in Szczecin. The isomerization of S-carvone was carried out in a glass reactor with a capacity of 25 cm^3^ under atmospheric pressure. A catalyst (15 wt% Ti-SBA-16, 0.75 g in relation to amount of S-carvone which amounted to 5 g) was introduced into a glass reactor equipped with a reflux condenser and a magnetic stirrer with a heating function The process of isomerization was carried out for 360 min at 210 °C. As a result of the isomerization reaction, carvacrol was obtained with a yield of 61 mol%, and the S-carvone conversion amounted to 85 mol%. The reaction mixture was then centrifuged to remove the catalyst. After removing the catalyst, the composition of the post-reaction solution was determined by gas chromatography. Thesolution contained carvacrol 60.4 wt % and S-carvone 14.88 wt%. In addition, the mixture contained by-products that were identified by GC-MS. Among the by-products identified in very small amounts in the post-reaction mixture after S-carvone isomerization were 4-(2-methylprop-2-en)phenol,1-methyl-4-(1-methyl-1-ene)-cylocyclohexan-2-one,1-methyl-4-(1-methyl-1-hydroxy-ethyl)cyclohex-1-en-2-one,4-hydroxy-4-methyl-pentan-2-one,2-(2-methylprop-2-en) phenol and carvone oxide.

### 2.3. Microbiological Tests

The microbiological tests were performed in the laboratory of Department of Chemical and Process Engineering West Pomeranian University of Technology in Szczecin. The antimicrobial properties of creams were tested against the Gram-negative bacteria *Escherichia coli* (ATCC 25922) and the Gram-positive bacteria *Staphylococcus epidermidis* (ATCC 49461), an opportunistic pathogenic yeast *Candida albicans,* and the dermatophytic fungus *Trichophyton rubrum* isolated from patients with immunodeficiency disorders from the Department of Chemical and Process Engineering DCPE collection. Additionally, selected mold fungi commonly found in air, such as N3 *Aspergillus niger* and ASM1 *Penicillium chrysogenum,* were tested. Both mentioned strains are in the *DCPE* collection and originated from fitness club air. Microorganisms came from the collection of the Department of Chemical and Process Engineering, West Pomeranian University of Technology Szczecin. The analyses were carried out by disc diffusion with some variations in the methodology [46]. To prepare the working culture of tested microorganism a preculture was made from the stock culture with recommended media for each microorganism. Table 1 lists the culture media used in this study.

Microorganism suspensions with 0.85% NaCl (Chempur, Poland) corresponding to 1–2 × 10^8^ CFU/mL for bacteria (0.5 in McFarland standard); 1–2 × 10^6^ CFU/mL for yeasts and 0.4–5.5 × 10^6^ CFU/mL for other fungi were prepared. Creams were stored at +4 °C and equilibrated to room temperature before use. Three paper discs (10 mm in diameter) were impregnated with 100 µL of fresh balm (approx. 0.01 g). After 24 h incubation (for bacteria and *C. albicans*) and 72 h for fungi on appropriate media and at the optimal microorganism temperature (Table 1) the formation of inhibition zones of growth was observed. Antimicrobial activities were determined by measuring the diameter of the inhibition zone. Control experiments with discs impregnated with cream base were carried out.

## 3. Results and Discussion

**Figure 2:**
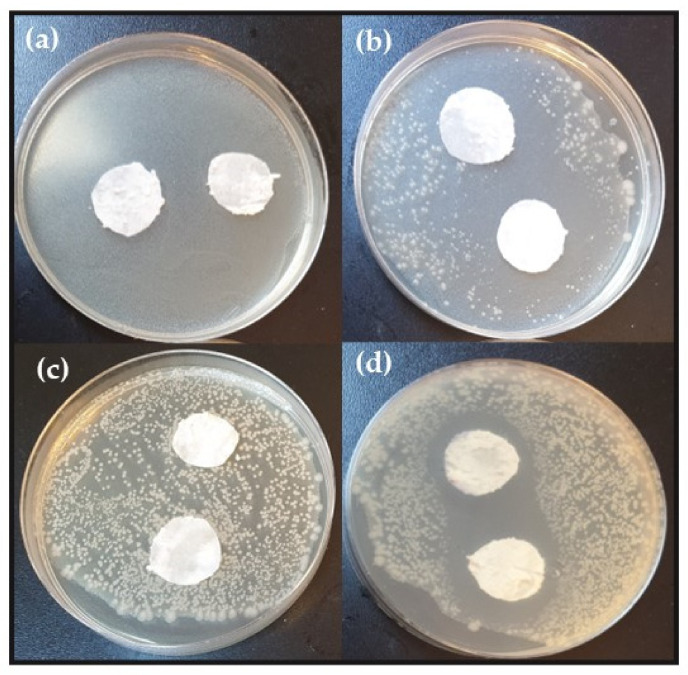
Effects of citral, nerol and carvacrol on the growth of *E. coli:* (**a**) 0.5% citral, (**b**) 2.0% nerol (**c**) 1% geraniol (**d**) 3.0% carvacrol.

These studies show that the antimicrobial properties of creams depended on the their composition and the concentrations of active substances against the target microorganisms. The results of the inhibition trials, performed by the disk diffusion method, are presented in Table 2 for the antibacterial tests, and in Table 3 for antifungal tests. The zone of inhibition around the discs impregnated with creams supplemented with nerol and linalool indicated bactericidal activity against both tested bacteria, while S-carvone, carvacrol, citral, and the mixture of isomerization products, showed bacteriostatic activity only against Gram-negative *Escherichia coli* (Table 2). Nerol and linalool in creams had a stronger inhibitory effect when they were applied in higher concentrations at 2 or 3 wt%. *Escherichia coli* was the most sensitive organism among those tested to inhibition by nerol at a concentration of 3 wt%. Growth of Gram-positive *S. epidermidis* was inhibited by nerol in concentrations from 1.0 to 3.0%, and by linalool concentration from 0.5 to 3.0%. Large, clear inhibition zones around disks impregnated with creams containing 2 wt% nerol and 3 wt% carvacrol, were observed (Figure 2). Results of antimicrobial activity of the tested substances are presented in Figure 2.

The pathogenic skin fungi *Candia albicans* and *Trichophyton rubrum* had high resistance against almost all tested additives. It was shown that introducing 3 wt% citral or carvacrol caused strong inhibition of the skin pathogenic fungi *T. rubrum* and *C. albicans*. Moreover, 2 wt% carvacrol and citral, or 3.0 wt% nerol and geraniol, hindered development of *C. albicans* (Table 2). The absence of activity for linalool was quite surprising since this monoterpene is known for its antifungal properties against *Candia* sp. and *Trichophyton* sp. However, as was shown by de Oliveira Lima, the linalool MIC values for *T. rubrum*, depend on mycelia stage of development and range from 256 mg/mL to 512 mg/mL [47].

The absence of inhibition zones indicated no effect of the tested creams supplemented with active compounds against the mould fungi *Aspergillus niger* and *Penicilium chrysogenum.* The sole exception was for creams with 3 wt% carvacrol or citral, for which small inhibition zones were noted (Table 3).

There was a statistically significant difference in the antimicrobial activity of tested compounds used in creams against bacteria and fungi. Almost all active substances showed more than twice the area of inhibition with bacteria compared to fungi. The low antimicrobial activity of linalool and S-carvone was surprising. Linalool is a monoterpene alcohol, and its enantiomers show high antimicrobial activity against several microorganisms, including bacteria *Escherichia coli, Staphylococcus aureus*, the yeast *Candida albicans,* the fungus *Botrytis cinerea*, and the protozoan *Plasmodium falciparum* [48]. Carvone is a monoterpene with antifungal properties even in a very low concentration (0.2%) against *Fusarium subglutinans*, *F. cerealis*, *Aspergillus tubingensis*, *F. verticillioides*, *Fusarium proliferatum*, *A. carbonarius*, *F. proliferatum*, *F. proliferatum*, *F. proliferatum, sporotrichioides*, *F. sporotrichioides* and *F. culmorum* [49]. Lack of activity in this study was probably due to theinactived enantiomer of linalool being used.

“Mixture” means the mixture obtained by isomerizing S-carvone.

## 4. Conclusions

Currently, increasing attention is being paid to chemical preservatives in cosmetics due to their demonstrated toxicity, carcinogenicity and teratogenicity. For these reasons, consumers look for cosmetics that contain only natural alternatives for the maintenance of product shelf-life. Our analysis of review articles shows that carvone, carvone, geraniol, linalool, citral and nerol possess antimicrobial and antioxidant activity. Many of these substances are generally recognized as safe substances (GRAS) and, therefore, could be used to prevent post-harvest growth of microorganisms that cause deterioration of cosmetics or are pathogenic microflora of the skin. The most important argument for using natural substances from plants is the lack of chemicals (preservatives) that are often found in the composition of the cosmetics. Natural cosmetics are products whose ingredients are at least 95% of natural origin, obtained by physical and chemical methods such as filtration, drying, distillation, pressing, extraction, isomerization or oxidation. There are many natural cosmetics on the market, but their quality and effectiveness are as important as their natural origins. This applies, in particular, to the efficacy of active substances i in the composition of cosmetics. Twenty-eight natural creams with attractive sensory qualities were tested against varied microorganisms in our studies. The concentration of tested active ingredients was very low, i.e., from 0.5 to 3% *v*/*v*. Only nerol and linalool were effective antibacterial compounds in creams against *E. coli* and *S. epidermidis* bacteria. Carvacrol, nerol, geraniol and citral at a 3% concentration had antifungal activity against the skin pathogens *C. albicans* and *T. rubrum.* Moreover, 3% carvacrol and citral poorly inhibited growth of the mould fungi *P. chrysogenum* and *A. niger*. Our preliminary results indicate that presence of natural compounds (terpenes) or their derivatives can significantly support the action of other preservatives and acts as preservative boosters. However, to evaluate if a cosmetic is well protected against microbial contamination the size of the pack should be taken into account, as well water content, water activity (aw) and the pH of the final product. In the future, we plan further research on the use of S-carvone and geraniol in creams, as well as their derivatives. Future research will focus on creams with higher levels of the tested compounds as well as post-reaction mixtures, that will be concentrated by distilling off the organic reagent before adding to the creams. In the case of isomerization of geraniol, the conditions for the isomerization process will be selected to reduce the number of products to a maximum two or three, to clearly define the influence of such mixtures on the microbiological effect of the creams. Additional research will be needed to answer the question of whether creams containing S-carvone, geraniol and their transformation products can have a healing effect on the skin and can be used in the future to treat cuts, allergies, eczema or atopic dermatitis. Future research should include stability tests of creams and their cytotoxicity. It would also be interesting to investigate the permeation ability of the active ingredients of creams through the skin, which could significantly increase the applicability of such creams. Since one of the compounds derived from geraniol is thumbergol, which has an anti-cancer effect [50], it would be interesting in the future to study the anti-cancer effect creams on the skin.

## Figures and Tables

**Table 1:** Incubation conditions and preculture and culture media used in antimicrobial tests.

Microorganism	Temperature Incubation (°C)	Preculture Media	Culture Media
*Escherichia coli*	37	Enrich Broth (BioMaxima S. A., Lublin, Poland)	Plate Count Agar (BioMaxima S.A., Poland)
*Staphylococcus epidermidis*	37	Brain Heart Infusion Broth (BioMaxima S. A., Poland)	Brain Heart Infusion Agar (BioMaxima S.A., Poland)
*Candida albicans* *Trichophyton rubrum*	30	Slants on Sabouraud Agar (BTL, Lodz, Poland)	Sabouraud Agar (BTL, Poland)
*Aspergillus niger* *Penicillium chrysogenum*	25	Slants on Malt Extract Agar, MEA (Merck, Warsaw, Poland)	Malt Extract Agar, MEA (Merck, Poland)

**Table 2:** Antibacterial test of creams containing Ss-carvone, geraniol, and derivatives.

Cream Supplemented with:	Bacteria		
	*E.coli*	*S. epidermidis*	
	**Isomerization of s-carvone**		
S-carvone 0.5%	18.5 ± 0.2	0 ± 0	
S-carvone 1.0%	25.0 ± 1.0	0 ± 0	
S-carvone 2.0%	7.0 ± 0.4 poor growth over the entire area of the plate	0 ± 0	
S-carvone 3.0%	>50 mm	0 ± 0	
carvacrol 0.5%	0 ± 0	0 ± 0	
carvacrol 1.0%	0 ± 0	0 ± 0	
carvacrol 2.0%	poor growth over the entire area of the plate	0 ± 0	
carvacrol 3.0%	>50 mm	0 ± 0	
mixture 0.5%	poor growth over the entire area of the plate >50 mm	0 ± 0	
mixture 1.0%		0 ± 0	
mixture 2.0%		0 ± 0	
mixture 3.0%		0 ± 0	
	**Isomerization of geraniol**		
geraniol 0.5%	0 ± 0	0 ± 0	
geraniol 1.0%	0 ± 0	0 ± 0	
geraniol 2.0%	0 ± 0	0 ± 0	
geraniol 3.0%	0 ± 0	0 ± 0	
nerol 0.5%	0 ± 0	0 ± 0	
nerol 1.0%	1.3 ± 0.2	0.3 ± 0.2	
nerol 2.0%	8.5 ± 0.5	0.7 ± 0.3	
nerol 3.0%	23.2 ± 1.3	1.8 ± 0.3	
linalool 0.5%	1.0 ± 0.2	1.0 ± 0.2	
linalool 1.0%	1.0 ± 0.2	1.0 ± 0.2	
linalool 2.0%	1.0 ± 0.2	1.0 ± 0.2	
linalool 3.0%	2.0 ± 0.1	2.0 ± 0.1	
citral 0.5%	poor growth over the entire area of the plate >50 mm	0 ± 0	
citral 1.0%		0 ± 0	
citral 2.0%		0 ± 0	
citral 3.0%		0 ± 0	
control	0 ± 0	0 ± 0	0 ± 0

**Table 3:** Antifungal tests of creams containing S-carvone and geraniol, and its isomerization products.

Cream Supplemented with:	Fungi			
	*C. albicans*	*T. rubrum*	*A. niger*	*P. chrysognum*
	Isomerization of S-carvone			
S-carvone 0.5%	0 ± 0	0 ± 0	0 ± 0	0 ± 0
S-carvone 1.0%	0 ± 0	0 ± 0	0 ± 0	0 ± 0
S-carvone 2.0%	0 ± 0	0 ± 0	0 ± 0	0 ± 0
S-carvone 3.0%	0 ± 0	0 ± 0	0 ± 0	0 ± 0
carvacrol 0.5%	0 ± 0	0 ± 0	0 ± 0	0 ± 0
carvacrol 1.0%	0 ± 0	0 ± 0	0 ± 0	0 ± 0
carvacrol 2.0%	1.0 ± 0.1	0 ± 0	0 ± 0	0 ± 0
carvacrol 3.0%	8.6 ± 1.4	5.4 ± 0.4	1.4 ± 0.3	1.3 ± 0.2
mixture 0.5%	0 ± 0	0 ± 0	0 ± 0	0 ± 0
mixture 1.0%	0 ± 0	0 ± 0	0 ± 0	0 ± 0
mixture 2.0%	0 ± 0	0 ± 0	0 ± 0	0 ± 0
mixture 3.0%	0 ± 0	0 ± 0	0 ± 0	0 ± 0
	**Isomerization of geraniol**			
geraniol 0.5%	0 ± 0	0 ± 0	0 ± 0	0 ± 0
geraniol 1.0%	0 ± 0	0 ± 0	0 ± 0	0 ± 0
geraniol 2.0%	0 ± 0	0 ± 0	0 ± 0	0 ± 0
geraniol 3.0%	1.0 ± 0.1	0 ± 0	0 ± 0	0 ± 0
nerol 0.5%	0 ± 0	0 ± 0	0 ± 0	0 ± 0
nerol 1.0%	0 ± 0	0 ± 0	0 ± 0	0 ± 0
nerol 2.0%	0 ± 0	0 ± 0	0 ± 0	0 ± 0
nerol 3.0%	1.3 ± 0.2	1.0 ± 0.1	0 ± 0	0 ± 0
linalool 0.5%	0 ± 0	0 ± 0	0 ± 0	0 ± 0
linalool 1.0%	0 ± 0	0 ± 0	0 ± 0	0 ± 0
linalool 2.0%	0 ± 0	0 ± 0	0 ± 0	0 ± 0
linalool 3.0%	0 ± 0	0 ± 0	0 ± 0	0 ± 0
citral 0.5%	0 ± 0	0 ± 0	0 ± 0	0 ± 0
citral 1.0%	0 ± 0	0 ± 0	0 ± 0	0 ± 0
citral 2.0%	2.0 ± 0.2	1.3 ± 0.2	0 ± 0	0 ± 0
citral 3.0%	7.6 ± 0.5	2.3 ± 0.6	1.2 ± 0.1	1.2 ± 0.2
control	0 ± 0	0 ± 0	0 ± 0	0 ± 0

## References

[1] Sun L., Zhang X., Zhang Y., Zheng K., Xiang Q., Chen N., Chen Z., Zhang N., Zhu J., He Q. (2019). Antibiotic-Induced Disruption of Gut Microbiota Alters Local Metabolomes and Immune Response. Front. Cell. Infect. Microbiol..

[2] Lee C. (2015). The antibiotic resistance crisis, part 1: Causes and threats. Pharm. Ther..

[3] Saad N.Y., Muller C.D., Lobstein A. (2013). Major bioactivities and mechanism of action of essential oils and their components. Flavour Fragr. J..

[4] Preedy V.R. (2016). Essential Oils in Food Preservation, Flavor and Safety.

[5] De Carvalho C.R., da Fonseca M.R. (2006). Carvone: Why and how should one bother to produce this terpene. Food Chem..

[6] Bailer J., Aichinger T., Hackl G., de Hueber K., Dachler M. (2001). Essential oil content and composition in commercially available dill cultivars in comparison to caraway. Ind. Crops Prod..

[7] Kamaleeswari M., Deeptha K., Sengottuvelan M., Nalini N. (2006). Effect of dietary caraway (*Carum carvi* L.) on aberrant crypt foci development, fecal steroids, and intestinal alkaline phosphatase activities in 1,2-dimethylhydrazine-induced colon carcinogenesis. Toxicol. Appl. Pharm..

[8] Husnu C., Baser K. (2008). Biological and pharmacological activities of carvacrol and carvacrol bearing essential oils. Curr. Pharm. Des..

[9] Hartmans K.J., Lenssen J.M., de Vries R.G. (1998). Use of talent (carvone) as a sprout growth regulator of seed potatoes and the effect on stem and tuber number. Potato Res..

[10] Esfandyari-Manesh M., Ghaedi Z., Asemi M., Khanavi M., Manayi A., Jamalifar H., Atyabi F., Dinarvand R. (2013). Study of antimicrobial activity of anethole and carvone loaded PLGA nanoparticles. J. Pharm. Res..

[11] Wah Y., Siow K.S., Yuen P., Gires U., Majlis B.Y. (2016). Plasma polymerized carvone as an antibacterial and biocompatible coating. Mat. Sci. Eng. C.

[12] McGeady P., Wansley D.L., Logan D.A. (2002). Carvone and perillaldehyde interfere with the serum-induced formation of filamentous structures in Candida albicans at substantially lower concentrations than those causing significant inhibition of growth. J. Nat. Prod..

[13] Raphael T.J., Kuttan G. (2003). Immunomodulatory activity of naturally occurring monoterpenes carvone, limonene, and perillic acid. Immunophar. Immunotoxicol..

[14] Retajczyk M., Wróblewska A., Szymańska A., Miądlicki P., Koren C., Michalkiewicz B. (2020). Synthesis, Characterization, and catalytic applications of the Ti-SBA-16 porous material in the selective and green isomerizations of limonene and S-carvone. Catalysts.

[15] Fan K., Li X., Cao Y., Qi H., Li L., Zhang Q., Sun H. (2005). Carvacrol inhibits proliferation and induces apoptosis in human colon cancer cells. Anticancer. Drugs.

[16] Ozen D., Uyanoglu M. (2018). Effect of carvacrol on IL-6/STAT3 pathway after partial hepatectomy in rat liver. Bratisl. Med. J..

[17] Canbeka M., Uyanoglua M., Bayramoglua G., Senturka H., Erkasapb N., Kokenc T., Uslud S., Demirustue C., Aralf E., Husnu K. (2008). Effects of carvacrol on defects of is chemia-reperfusion in the rat liver. Phytomedicine.

[18] Rattanachaikunsopon P., Phumkhachorn P. (2010). Assessment of factors influencing antimicrobial activity of carvacrol and cymene against Vibrio cholerae in food. J. Biosci. Bioeng..

[19] Nostro A., Roccaro A.S., Bisignano G., Marino A., Cannatelli M.A., Pizzimenti F.C., Luigi C.P., Procopio F., Blanco A.R. (2007). Effects of oregano, carvacrol and thymol on Staphylococcus aureus and Staphylococcus epidermidis biofilms. J. Med. Microbiol..

[20] Khan I., Bahuguna A., Kumar P., Bajpai V.K., Kang S.C. (2017). Antimicrobial Potential of Carvacrol against Uropathogenic Escherichia coli via membrane disruption, depolarization, and reactive oxygen species generation. Front. Microbiol..

[21] Vardar-Unlu G., Yagmuroglu A., Unlu M. (2010). Evaluation of in vitro activity of carvacrol against Candida albicans strains. Nat. Product Res..

[22] Calo J.R., Crandall P.G., O’Bryan C.A., Ricke S.C. (2015). Essential oils as antimicrobials in food systems—A review. Food Control.

[23] Papachristos D., Karamanoli K., Stamopoulos D.C., Menkissoglu-Spiroudi U. (2004). The relationship between the chemical composition of three essential oils and their insecticidal activity against Acanthoscelides obtectus (Say). Pest Manag. Sci..

[24] Chen W., Viljoen A.M. (2010). Geraniol—A review of a commercially important fragrance material. South Afr. J. Bot..

[25] Hadian Z., Maleki M., Feizollahi E., Alibeyk S., Saryazdi M. (2020). Health aspects of geraniol as a main bioactive compound of Rosa damascena Mill: A systematic review. Electron. Physician.

[26] Mączka W., Wińska K., Grabarczyk M. (2020). One hundred faces of geraniol. Molecules.

[27] Fajdek-Bieda A., Wróblewska A., Miądlicki P., Tołpa J., Michalkiewicz B. (2021). Clinoptilolite as a natural, active zeolite catalyst for the chemical transformations of geraniol. Reac. Kinet. Mech. Catal..

[28] Yamada A.N., Grespan R., Yamada A.T., Silva E.L., Silva-Filho S.E., Damiao M.J., de Oliveira Dalalio M.M., Bersani-Amado C.A. (2013). Anti-inflammatory activity of *Ocimum americanum* L. essential oil in experimental model of zymosan-induced arthritis. Am. J. Chin. Med..

[29] Djenane D., Aider M., Yanguela J., Idir L. (2012). Antioxidant and antibacterial effects of Lavandula and Mentha essential oils in minced beef inoculated with E. coli O157:H7 and S. aureus during storage at abuse refrigeration temperature. Meat. Sci..

[30] Quintans-Junior L.J., Barreto R.S., Menezes P.P., Almeida J.R. (2013). β-Cyclodextrin-complexed (-)-linalool produces antinociceptive effect superior to that of (-)-linalool in experimental pain protocols. Basic Clin. Pharmacol. Toxicol..

[31] Phillips C.A., Gkatzionis K., Laird K., Score J. (2012). Identification and quantification of the antimicrobial components of a citrus essential oil vapor. Nat. Prod. Commun..

[32] Aprotosoaie A.C., Hancianu M., Costacheb I.I., Mirona A. (2014). Linalool: A review on a key odorant molecule with valuable biological properties. Flavour Fragr. J..

[33] Kamatou G.P., Viljoen A.M. (2008). Linalool—A review of a biologically active compound of commercial importance. Nat. Prod. Commun..

[34] Lis-Balchin M. (1997). Essential oils and ‘aromatherapy’: Their modern role in healing. J. Royal Soc. Promot. Health.

[35] Lapczynski A., Foxenberg R.J., Bhatia S.P., Letizia C.S., Api A.M. (2008). Fragrance material review on nerol. FCT.

[36] Costa Marques T.H., Leonildes B., Gomes C., Branco Marques M., dos Santos Lima D., Santos Siqueira H.D., Damasceno Nogueira Neto J., do Socorro Boavista Gomes Castelo Branco M., Araújo de Souza A., Pergentino de Sousa D. (2013). Evaluation of the neuropharmacological properties of nerol in mice. World J. Neurosci..

[37] Ammar A.H., Bouajila J., Lebrihi A., Mathieu F., Romdhane M., Zagrouba F. (2012). Chemical composition and in vitro antimicrobial and antioxidant activities of Citrus aurantium L. flowers essential oil (Neroli oil). Pak. J. Biol. Sci..

[38] Consolini A.E., Berardi A., Rosella M.A., Volonté M. (2011). Antispasmodic effects of Aloysia polystachya and A. gratissima tinctures and extracts are due to non- competitive inhibition of intestinal contractility induced by acethylcholine and calcium. Rev. Bras. Farmacogn..

[39] Kuwahara Y., Suzuki H., Matsumoto K. (1983). Pheromone study on acarid mites. XI. Function of mite body as geometrical isomerization and reduction of citral (the alarm pheromone) Carpoglyphus lactis. Appl. Entomol. Zool..

[40] Bouzenna H., Hfaiedh N., Giroux-Metges M.A., Elfeki A., Talarmin H. (2017). Biological properties of citral and its potential protective effects againstcytotoxicity caused by aspirin in the IEC-6 cells. Biomed. Pharmacother..

[41] Espina L., Daniel B., Patricia A., García-Gonzalo D., Rafael P. (2017). Potential use of carvacrol and citral to inactivate biofilm cells and eliminate biofouling. Food Control.

[42] Yuxiang Z., Wei J., Chen H., Song Z., Guo H., Yuan Y., Yue T. (2020). Antibacterial activity of essential oils against Stenotrophomonas maltophilia and the effect of citral on cell membrane. LWT.

[43] Kang S., Li X., Xing Z., Liu X., Bai X., Yang Y., Guo D., Xia X., Zhang C., Shi C. (2022). Antibacterial effect of citral on yersinia enterocolitica and its mechanism. Food Control.

[44] Cristiane de Bona da S. (2008). Antifungal activity of the lemongrass oil and citral against Candidas. Braz. J. Infect. Dis..

[45] Cai R., Hu M., Zhang Y., Niu C., Yue T., Yuan Y., Wang Z. (2019). Antifungal activity and mechanism of citral, limonene and eugenol against Zygosaccharomyces rouxii. LWT.

[46] Clinical and Laboratory Standards Institute (2009). Approved standard M2-A10.

[47] Pucci M., Raimondo S., Zichittella C., Tinnirello V., Corleone V., Aiello G., Moschetti M., Conigliaro A., Fontana S., Alessandro R. (2020). Biological properties of a citral-enriched fraction of Citrus limon essential oil. Foods.

[48] De Oliveira Lima M.I., Araújo de Medeiros A.C., Souza Silva K.V., Cardoso G.N., de Oliveira Lima E., de Oliveira Pereira F. (2017). Investigation of the antifungal potential of linalool against clinical isolates of fluconazole resistant Trichophyton rubrum. J. Mycol. Méd..

[49] Özek T., Tabanca N., Demirci F., Wedge D.E., Baser K.H.C. (2010). Enantiomeric distribution of some linalool containing essential oils and their biological activities. Rec. Nat. Prod..

[50] Abi-Ayad M., Abi-Ayad F.Z., Lazzouni H.A., Rebiahi S.A., Ziani_Cherif C., Bessiere C. (2011). Chemical composition and antifungal activity of Aleppo pine essential oil. J. Med. Plant Res..

